# Merkel Cell Carcinoma with Lymphoepithelioma-Like Pattern: A Case Report of an Exceedingly Rare Variant of Merkel Cell Carcinoma with Lymph Node Metastases at Presentation

**DOI:** 10.1155/2011/840575

**Published:** 2011-09-06

**Authors:** Soumaya Ben Abdelkrim, Abdelmajid Dhouibi, Adnène Moussa, Rim Hadhri, Leila Njim, Khalifa Mighri, Abdelfatteh Zakhama

**Affiliations:** ^1^Department of Pathology, Fattouma Bourguiba University Hospital, 1st June Avenue, Monastir 5000, Tunisia; ^2^Department of Otorhinolaryngology, Tahar Sfar University Hospital, Mahdia 5111, Tunisia

## Abstract

Merkel cell carcinoma (MCC) or primary neuroendocrine carcinoma of the skin is a rare neoplasm with aggressive behavior. Primary lymphoepithelioma-like (LEL) carcinoma of the skin is a recently described exceptional tumor, with a relatively good prognosis, and is characterized by a neoplastic epithelial component associated with a dense lymphoid stroma. Rarely, MCC shows a marked lymphocytic host response or can even mimic a LEL carcinoma. We report a new case of MCC mimicking an LEL carcinoma in a 72-year-old male; the diagnosis of MCC was made on the basis of the morphology and immunohistochemical findings. We present through this case an exceptional pattern of MCC which can be misleading, and we insist on differential diagnoses.

## 1. Introduction

MCC or primary neuroendocrine carcinoma of the skin is a rare and aggressive neoplasm with a high metastatic potential [1]. Primary LEL carcinoma of the skin is a very uncommon and recently recognized tumor with a favorable outcome [2, 3]. Marked lymphocytic infiltrate is an uncommon feature in MCC [4]. We describe here an exceedingly rare pattern of MCC mimicking LEL carcinoma diagnosed in a 72-year-old male on the basis of the primitive tumor and lymph nodes' metastases morphology and immunohistochemical findings.

## 2. Case Presentation

**Figure 1:**
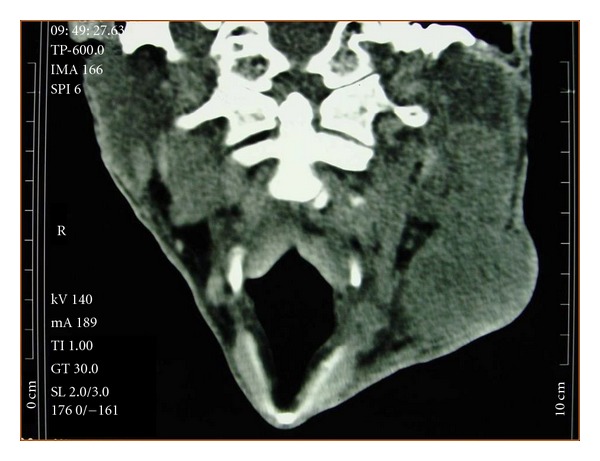
Computed tomography scan showing the lymph nodes metastases of Merkel cell carcinoma.

**Figure 2:**
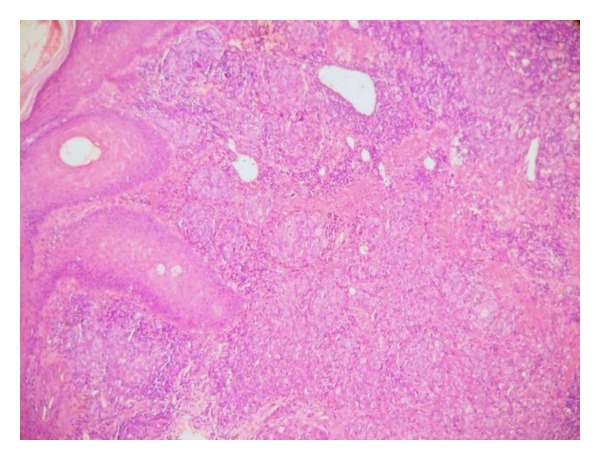
The dermis shows a proliferation of lobules in a lymphoid background (H&E, original magnification ×40).

**Figure 3:**
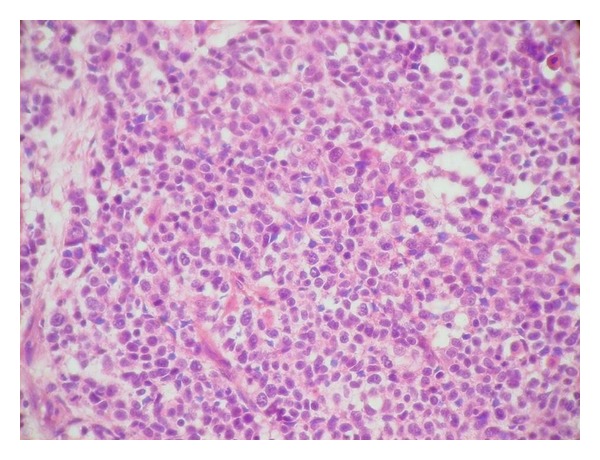
Tumor cells are pleomorphic with vesicular nuclei and prominent nucleoli (H&E, original magnification ×200).

**Figure 4:**
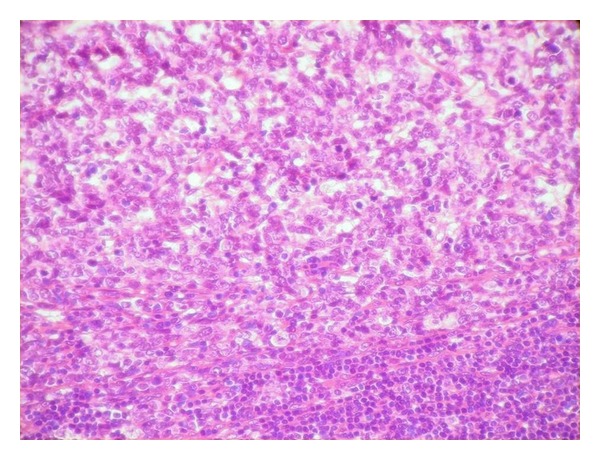
The lymph node is massively infiltrated by a proliferation of monotonous basophilic cells (H&E, original magnification ×100).

**Figure 5:**
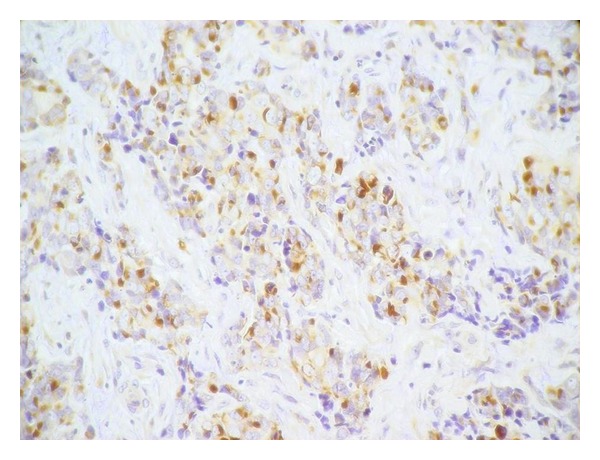
Tumor cells are positive for neurofilament (immunohistochemistry ×400).

**Figure 6:**
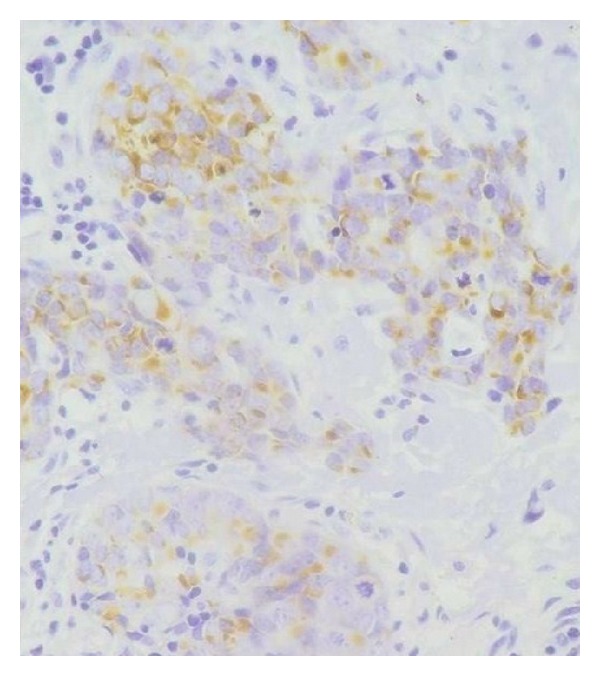
Tumor cells are positive for cytokeratin 20 with a dot-like staining (immunohistochemistry ×400).

A 72-year-old man had a two-month history of a left-sided, slowly enlarging, painful mass of the cervical region. His medical history did not highlight any significant evidence. On examination, the mass was fixed to the deep plan, hard in consistency, measuring 4 cm in diameter with external signs of inflammation. The rest of examination revealed a skin ulcerated tumor of the forehead measuring 1.5 cm which appeared one month ago. The remaining systemic examination did not reveal any coexistent lesions. Cervical ultrasonography and computed tomography scan suggested enlarged cervical lymph nodes (Figure 1). Random biopsies from the nasopharyngeal mucosa were normal. The patient underwent a cervical lymph node biopsy and an excision of the frontal lesion. The excised cutaneous specimen showed a dermal carcinomatous proliferation with features of LEL carcinoma, characterized by a nonneoplastic prominent lymphocytic infiltrate intermingled with a poorly differentiated epithelial proliferation with syncytial appearance (Figures 2 and 3). Cervical lymph node was massively infiltrated by a dense carcinomatous proliferation suggesting neuroendocrine differentiation, and it was made of monomorphous small basophilic cells with a very fine chromatin and minimal cytoplasm dispersed in a scanty stroma (Figure 4). Frequent mitotic figures were found. Immunohistochemical stains of the cutaneous tumor and lymph node metastases showed immunoreactivity for neurofilament (Figure 5), chromogranin, synaptophysin, and a characteristic dot-like perinuclear staining for cytokeratin 20 (Figure 6). The tumor did not express TTF1. On the basis of these findings, the diagnosis of MCC with ipsilateral cervical lymph node metastasis was made. The patient was going to undergo radiotherapy and died 3 months after the diagnosis.

## 3. Discussion

Primary LEL carcinoma of the skin is distinctly uncommon; since its initial description by Swanson et al. in 1988 [5], only 47 cases have been documented to date [3]. The first Tunisian case was reported only in 2006 [6]. Histologically, LEL carcinoma is indistinguishable from undifferentiated nasopharyngeal carcinoma—which is much more common—or other LEL carcinomas that develop in various parts of the body. Therefore, to confirm the diagnosis of primary LEL carcinoma of the skin, metastatic nasopharyngeal carcinoma to the skin should be eliminated by examination of the upper aerodigestive tract with endoscopy and even random biopsy of the nasopharynx [1]. Unlike its nasopharyngeal counterpart, primary LEL carcinoma of the skin has a relatively good prognosis and among the 47 previously reported cases, only 2 patients developed lymph node metastases and had a fatal course [3]. Classically, this cancer appears as a progressively growing nodule, rarely ulcerated, affecting preferentially elderly patients and occurring mostly in the head and neck region [2]. This clinical presentation is also valid for MCC, a neoplasm with an aggressive behavior, first described in 1972 by Toker [7] under the term of “trabecular carcinoma of the skin”. Histologically, this tumor has a solid or a trabecular growth pattern and is made of cells with scanty cytoplasm and round nuclei with granular chromatin and several nucleoli [4]. Divergent differentiations in MCC have been described in isolated case reports such as squamous differentiation and less frequently glandular, melanocytic, eccrine, rhabdomyoblastic, neuroblastic, leiomyosarcomatous, or lymphoepithelial-like differentiation [8]. The latter is extremely rare, and after a review of the literature, we believe that we report here the third case of MCC mimicking LEL carcinoma. This entity was described for the first time by Rosso et al. in 1998 [9], and the lymphoepithelial-like differentiation was predominant with only a small percentage of a classic neuroendocrine contingent. In our case, histological findings of the skin tumor did not suggest MCC. The second reported case was documented, two years later, by Rios-Martin et al. [10]. Some cases of MCC with a marked lymphocytic host response suggesting a lymphomatous proliferation have been reported. Bastian et al. [1] have described an MCC with predominant lymphocytic component and several lymphoid follicles hiding the scattered neoplastic cells, and the lesion was simulating a lymphocytoma or a follicular lymphoma. Two other similar cases of an MCC with a follicular lymphocytic infiltrate were recently described [4]. Kasami et al. [11] have reported a new entity that they called large cell neuroendocrine carcinoma of the skin; the two cases they described showed an abundant lymphocytic infiltration making the differential diagnosis difficult with MCC mimicking LEL carcinoma and especially an LEL carcinoma of the skin. In this proposed new entity, tumor cells are larger and more pleomorphic than those of conventional MCC, and the characteristic perinuclear immunostaining with cytokeratin 20 is not found. The expression of one neuroendocrine marker is sufficient to make the diagnosis of large cell neuroendocrine carcinoma of the skin. Finally, let us remember that a dense lymphocytic infiltrate can be seen in case of spontaneous regression of MCC [12].

It is worth underlining that when dealing with a cutaneous tumor suggesting LEL carcinoma, we should eliminate metastatic nasopharyngeal carcinoma to the skin and an MCC. Immunohistochemistry has gained a great importance in establishing the diagnosis, showing a typical perinuclear dot-like positivity with cytokeratin 20 in MCC and the absence of immunostaining with neuroendocrine markers in LEL carcinoma [3].

## 4. Conclusion

An abundant lymphocytic infiltrate may be rarely seen in MCC, and it does not seem to improve the prognosis of this aggressive neoplasm. An MCC can exceptionally mimic LEL carcinoma of the skin by the lymphocytic infiltrate and the cells' morphology. This entity has been recently described as a large cell neuroendocrine carcinoma and needs to be validated. Anyway, neuroendocrine markers should be used in case of a cutaneous tumor rich in lymphocytes even if the morphology is typical of an LEL carcinoma. In fact, recognition of this variant of MCC is important, because it can easily be mistaken for LEL carcinoma resulting in suboptimal treatment.
